# Multifunctional dietary interventions, low-grade inflammation and cardiometabolic profile: a scoping review

**DOI:** 10.3389/fimmu.2024.1304686

**Published:** 2024-02-27

**Authors:** Hugo Hornero-Ramirez, Adrien Aubin, Marie-Caroline Michalski, Sophie Vinoy, Cyrielle Caussy, Julie-Anne Nazare

**Affiliations:** ^1^ Centre de Recherche en Nutrition Humaine Rhône-Alpes, CarMeN lab, Univ-Lyon, INSERM, INRAe, Claude Bernard Lyon1 University, Centre Hospitalier Lyon Sud, Hospices Civils de Lyon, Pierre Bénite, France; ^2^ Département Endocrinologie, Diabète et Nutrition, Hôpital Lyon Sud, Pierre-Bénite, Hospices Civils de Lyon, Pierre Bénite, France; ^3^ Nutrition Research, Paris Saclay Tech Center, Mondelez International R&D, 91400, Saclay, France

**Keywords:** bioactive compound, food synergy, oxidative stress, Mediterranean, Nordic, portfolio, cardiometabolic profile, human

## Abstract

**Background:**

Growing evidence highlights the significant impact of diet to modify low-grade inflammation closely linked to cardiometabolic profile. Multifunctionnal diets, combining several compounds have been shown to beneficially impact metabolic parameters.

**Objective:**

This study synthesizes the knowledge on the impact of RCTs combining dietary multifunctional compounds on low-grade inflammation in humans. We investigate whether the effects of dietary multifunctional interventions on inflammatory markers were parallel to alterations of cardiometabolic parameters.

**Methodology:**

We considered both the integrated dietary interventions (ID, i.e. global diets such as Mediterranean, Nordic…) and the dietary interventions based on selected bioactive mix (BM) compounds, in healthy individuals and those at cardiometabolic risk. Out of 221 screened publications, we selected 27 studies: 11 for BM (polyphenols and/or omega-3 fatty acids and/or antioxidants and/or dietary fiber) and 16 for ID (Mediterranean, paleo, Nordic, Dietary Approaches to Stop Hypertension (DASH) diet…).

**Results:**

ID studies reflected significant improvements in inflammatory markers (CRP, IL-6, IL-10, IL-1b), concomitantly with beneficial changes in metabolic parameters. In BM studies, pronounced effects on low-grade inflammatory markers were observed, while improvements in metabolic parameters were not consistent. Both types of studies suggested a favorable impact on oxidative stress, a factor closely linked to the inflammatory profile.

**Conclusion:**

Our findings showed that multifunctional RCT diets have differential role in managing low-grade inflammation and cardiometabolic health, with a large heterogeneity in explored inflammatory markers. Further research is imperative to elucidate the link between low-grade inflammation and other cardiometabolic risk factors, such as intestinal inflammation or postprandial inflammatory dynamics, aiming to attain a comprehensive understanding of the mechanisms involved in these processes. These future investigations not only have the potential to deepen our insights into the connections among these elements but also pave the way for significant advancements in the prevention and management of conditions related to the cardiovascular and metabolic systems.

## 1 Introduction

The increasing prevalence of cardiometabolic diseases (CDs), such as obesity, type 2 diabetes mellitus (T2DM), cardiovascular diseases, and metabolic dysfunction-associated fatty liver disease (MAFLD) represents a global public health challenge. Chronic low-grade inflammation is considered as a common underlying pathophysiological feature of these chronic diseases (1). Low-grade inflammation reflects a sustained and subtle inflammatory response occurring at a low level in the body over an extended duration. It is characterized by the production of pro-inflammatory cytokines and immune cell activation, resulting from a multifaceted interplay of inflammatory processes originating from a multitude of tissues (2). This persistent inflammatory state has been implicated in the pathogenesis of numerous chronic conditions, such as cardiovascular disease and diabetes (3). Moreover, it has been emphasized that the interaction between dietary compounds and the gut is a critical component of immunomodulatory effects, identified as both a potential cause and therapeutic strategy to help managing low-grade inflammation and metabolic abnormalities (3). Dietary patterns have been scientifically associated with variations in the inflammatory profile in particular with low-grade inflammation markers.

Liselot Koelman et al. showed in a meta-analysis the beneficial effects of certain dietary patterns, notably Mediterranean diet inducing the reduction of inflammatory biomarkers such as IL-6, IL-1b and CRP, reflecting an impact on several immunometabolic pathways (4). Research on the effects of food components on low-grade inflammation includes n–3 fatty acids, dietary fiber, polyphenols, antioxidants and a number of trials have shown positive effects on IL-6, TNF-a, CRP. Garcia-Arellano et al. demonstrated in the PREDIMED study that a Mediterranean diet characterized by higher consumption of foods such as fruits, vegetables and whole grains characterized by a low inflammatory potential (low-inflammatory index, an assigned score to different foods and nutrients according to their inflammatory properties) was associated with a reduced risk of cardiovascular disease (5). Beyond the well documented association between dietary patterns and inflammatory profile, dietary interventions which target low-grade inflammation have led to divergent results. Such discrepancies could be attributed to differences in tested dietary compounds or tested mix, in chosen inflammatory markers of interest or in study design (6, 7). Low-grade inflammation involves various different organs, cellular mediators and biochemical pathways and no consensus to date has been reached to determine the best marker or markers’ signature of low-grade inflammation (4, 8). Accordingly, previous works have defined the combination of several different food ingredients with proven health effect within the same diet as multifunctional interventions (9). The objective of this review is to synthesize the knowledge on the impact of RCTs (randomized controlled trial) dietary multifunctional interventions on low-grade inflammation in healthy and at cardiometabolic-risk subjects in relation to cardiometabolic profile. We considered both the integrated diet interventions (ID, global dietary patterns such as Mediterranean, Nordic…) and the dietary interventions based on selected bioactive mixes (BM), while analyzing all the different biomarkers assessed in RCTs as surrogates of low-grade inflammatory profile. Concomitantly, we investigated whether the effects of dietary multifunctional interventions on inflammatory markers were associated to alterations of other metabolic parameters such as lipidic, glycemic, and anthropometric parameters.

### 1.1 Methods

#### 1.1.1 Literature search strategy

A bibliographic research was conducted using an electronic search performed on PubMed/Google Scholar that includes all original research articles based on RCTs, parallel or crossover design, written in English, published after 2003. The search terms criteria were: [(intervention) AND (bioactive compounds OR anti-inflammatory OR low-grade inflammation OR inflammatory OR inflammatory markers OR polyphenol OR PUFA OR Portfolio OR Bioactive foods OR Whole Diet OR Multifunctional OR bioactive mix)] AND (inflammation) AND (dietary) AND (humans) NOT (animals). The final search was carried out on June 14, 2023.

#### 1.1.2 Inclusion and exclusion criteria

Selected studies involved adult male or female patients with a body mass index (BMI) between 18 and 40 kg/m². Only studies that included at one inflammatory marker (plasma/serum) as described as measures of analyses were included in the review with secondary metabolic outcomes such as lipidic and glycemic [glycaemia, insulin, non-esterified fatty acids (NEFA), triglycerides, adiponectin, Apolipoprotein B, and total, HDL, LDL cholesterol, HOMA, short chain fatty acids (SCFA)], anthropometric (body composition, weight, height, waist circumference, hip circumference),cardiac (blood pressure, tension) and gut markers (metagenomics).

**Figure 1:**
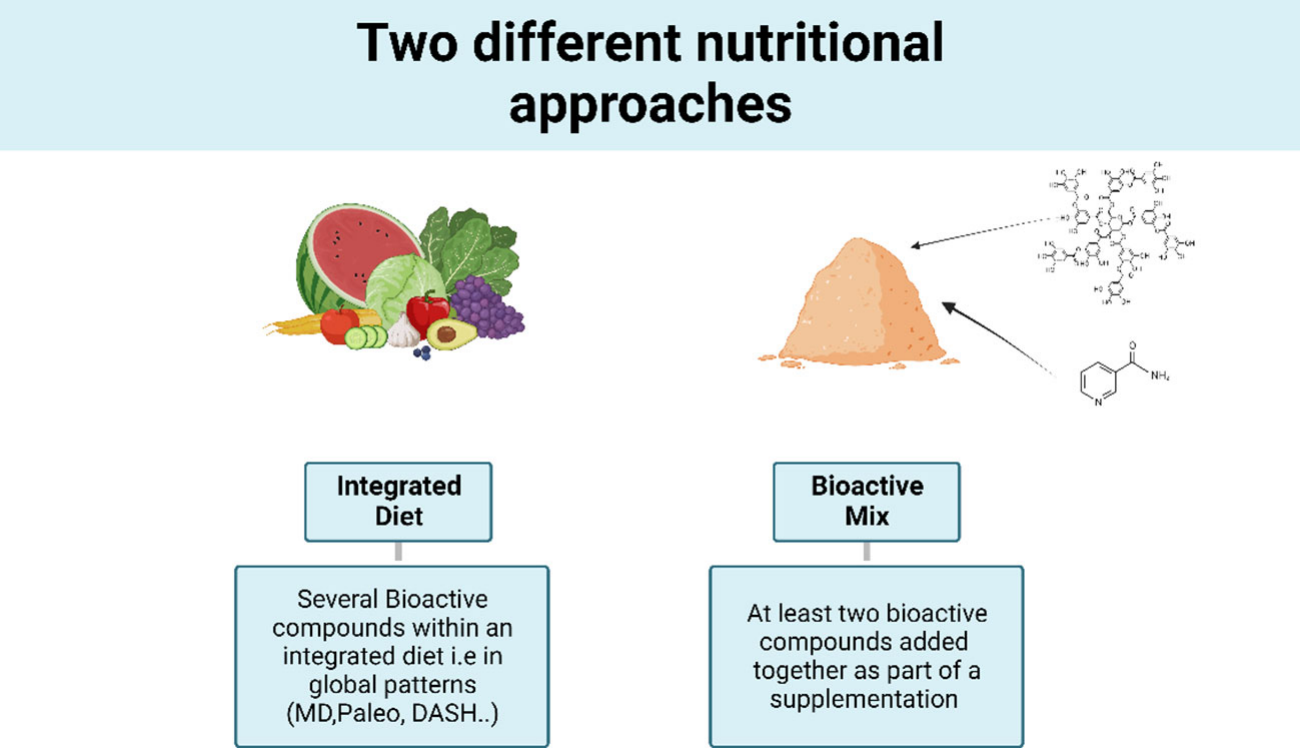
Multifunctional dietary interventions.

The search criteria for biomarkers of inflammation encompassed terms such as “low-grade inflammation,” “inflammatory,” “inflammatory markers,” and “oxidative stress.” In addition, anthropometric, metabolic criteria, or gut microbiota measurements were considered if the article included at least one inflammatory criterion. Included studies encompasses RCTs involving an integrated diet (ID), i.e., global dietary patterns, or RCTs investigating at least two bioactive compounds added together as part of a supplementation (bioactive mix, BM) (**Figure 1**).

Exclusion criteria were: i) design non-randomized trials, non-controlled trials, observational studies; ii) Population: studies carried out on animal models or *in vitro*, studies carried out on subjects with BMI> 40 kg.m², with cardiovascular disease (CVD), inflammatory pathology [e.g. rheumatoid arthritis, nonalcoholic steato-hepatitis (NASH)] or pathology affecting glucose metabolism (type 1 and 2 diabetics) or a specific group of subjects (pregnant women, breastfeeding women, study population <18 years, mental disorders). The study included both ad libitum and strict nutrition interventions and excluding caloric restriction, as well as research involving bioactive mixtures or integrated dietary approaches. However, studies focusing solely on the impact of a single bioactive compound were not considered. Additionally, studies examining physical activity or caloric restriction protocols were excluded if there were discrepancies between the placebo and test interventions.

#### 1.1.3 Selection

**Figure 2:**
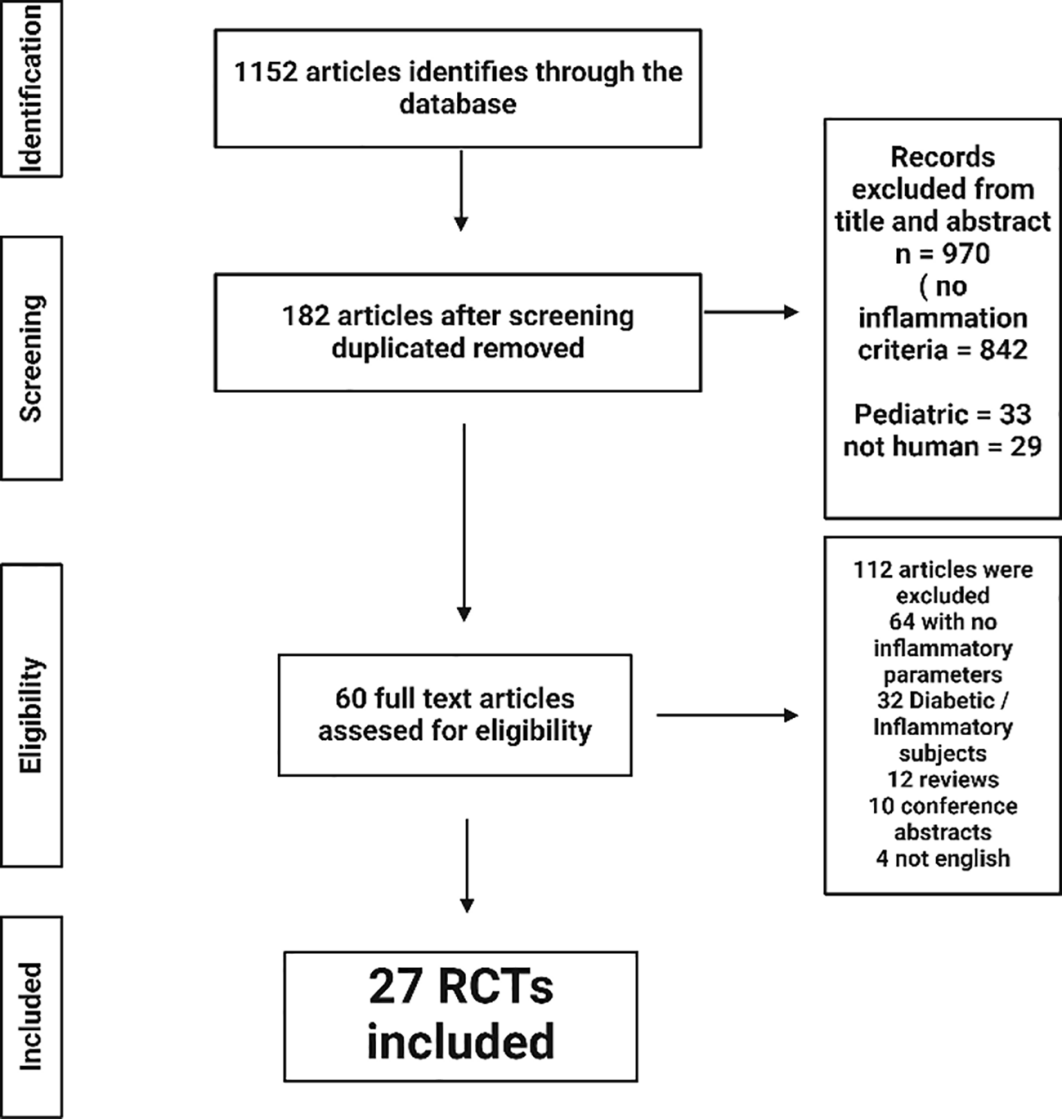
Diagram illustrating the literature search and study selection.

Two investigators (H.H and A.A) independently assessed each article during each step of the article retrieval process from the Pubmed and Google scholar databases (**Figure 2**). First, the titles of the articles were screened, as well as the abstracts to identify articles which potentially meet the inclusion search inclusion criteria. Second, the full texts were retrieved and screened to verify eligibility for inclusion based on inclusion and exclusion criterion. Any disagreements were resolved by discussion among the investigators and included discussion with a third researcher (J.N) until consensus was reached.

Finally, for each selected article, a data extraction form was used to collect information on the article (authors, title, source, year), the study population and baseline measurements, the study design, the study duration, the type of intervention, the amount of bioactive compounds, the outcome measures, and main findings.

## 2 Results

### 2.1 Study selection

The first stage of the research process identified 1152 items. By analyzing the titles and abstracts, 182 articles were identified after removing the duplicate articles. Sixty-four articles were excluded because of the absence of inflammatory markers assessed in the study, 32 because the type of subjects (mainly diabetics) or inflammations diseases (rheumatoid polyarthritis). Of the remaining articles, 32 were journal articles, 10 were abstracts of scientific presentations at congresses and 4 corresponded to articles in languages other than English.

Among these, a further selection was done after selecting by study design and exclusion criteria, 27 RCTs from the 60 that were eligible by title and abstract. Common reasons for exclusion were inclusion criteria not met; languages other than English or French; duplicates and unavailability of results on inflammation criteria. The two types of studies will be analyzed separately, first the integrated diet (ID) studies and then the bioactive mix (BM). Of the 27 interventional studies, 16 were integrated diets, which are diets that include all foods present in a day and include at least two bioactive components that have shown effects on inflammation. Concerning the 11 selected BM studies; these articles included the intake of a test product with a minimum of two bioactive compounds within the same matrix. Full details of each study are presented in **Table 1** for the ID and in **Table 2** for the BM, summarizing the descriptions and types of interventions with the amount of the main bioactive compounds. The analysis comprehensively explored various parameters beyond inflammatory markers, encompassing metabolic factors. In addition, it looked at other metabolic markers such as the composition of the gut microbiota, recognizing its recently unveiled role in a wide range of physiological processes and diseases.

**Table 1:** Integrated diet intervention studies investigated the impact on systemic inflammation, oxidative stress and cardiometabolic risk markers.

References	Design	BMI	Treatment	n	Length (weeks)	Proportion of male subjects	Age	Components of the dietary intervention	Response variables	Positive (↑). inverse (↓). and no (↔) statistically significant associations (pvalue)/Pourcentage change Δ placebo vs enriched
Adamsson V et al., 2011 (10)	Parallel non blinded	26	CD	42	6	34%	53	Low fiber/PUFAs	CRP LDL HDL ApoB/ApoA1 Insulin Weight loss Systolic Blood Pressure (BP)	↔ (p=0.4)/Δ6% vs -20% ↓ (p < 0.05) ↓ (p < 0.05) ↓ (p < 0.05) ↓ (p < 0.05) ↓ (p < 0.05) ↓ (p < 0.05)
			ND	44		38%		DF (g/MJ) >3 Beta Glucan >3g/day PUFAs (5–10% of energy)		
Fritzen et al., 2015 (11)	Parallel non blinded	31	Average Danish Diet	43	26	32%	42	Average Danish Diet	CRP HOMA TNF-a	↔ (NS) Δ 73% vs -41% (women) Δ -9% vs 15% (men) ↓ (p<0.001) ↔ (NS) Δ7% vs -41% (women) Δ -5% vs 7% (men)
			New ND	21				Berries, cabbages, root vegetables and legumes potatoes fresh herbs, wild plants and mushrooms nuts. whole grain. meats from livestock and game. fish and shellfish and seaweed		
Uusitupa et al., 2013 (12)	Parallel single blinded	31	ND	93	18-24	30%	54	Nordic Diet 6,8 (% energy) of PUFAs 34,7g of DF	IL-1 RA IL-6 IL-10 Il-1b non-HDL ApoB/ApoA1	↓ (p=0.00053) Δ 27% vs -0.5% ↔ (p=0.44)/Δ5%vs 10% ↔ (p=0.96)/Δ6%vs 3% ↔ (p=0.42)/Δ-4% vs 15% ↓ (p = 0.04) ↓ (p = 0.025)
			CD	70		27%		Control Diet 4,4 (% energy) of PUFAs 15,9g of DF		
Poulsen et al., 2014 (13)	Parallel non blinded	30	CD	47	24	29%	42	Control Diet (habitual Danish Diet)	CRP	↓ (p=0.43)
			New ND	73			41	New nordic Diet	Weight loss/BP	↓ (p<0.01)
Kirwan et al., 2016 (14)	Cross over double blinded	33	RD	33	8	18%	39	refined grain diet 23g of saturated fat 21g of DF	hsCRP Pulse Pressure	↔ (p=0.06) Δ-39% vs 18% ↓ (p=0.03)
			WG					whole grain diet 21g of saturated fat 29g of DF		
Vanegas et al., 2017 (15)	Parallel	26	RG group	40	6	60%	54	refined grain diet	IL-10 IL-6 IL-17 TNF-a TGF-b SCFA Production	↔ ns/Δ -43% vs 71% ↔ ns/Δ -15% vs -28% ↔ ns/Δ 84% vs 14% ↔ ns: Δ20% vs -5% ↔ ns/Δ300% vs 85% ↓ (p<0.05)
			WG	41			55	whole grain diet		
Tovar et al., 2014 (16)	Cross over	28	WG	46	4	0%	61	47g of DF 7g of PUFAs	CRP LDL ApoB/GGT Diastolic BP	↔ (p=0.47)/Δ-2% vs -5% ↓ (p=0.001) ↓ (p=0.001) ↓ (p<0.05)
			CD					Control Diet		
Freese et al., 2004 (17)	Parallel	22	TI	18	6	27%	25	P1 rich in PUFAs (10,9%) low in vegetables (167g/10 MJ) low in fresh fruits (54g/10MJ)	P-selectin ICAM-1 CRP Ox-LDL	↔ (p=0.819)/Δ-5% vs -8% ↔ (p=0.318) Δ5.14 vs -3.8 ↔ (p=0.264) Δ-16.08% vs -43.7% ↔ (p=0.668)
				20		25%	26	P2 rich in PUFAs (11,1%) high in vegetables (440g/10MJ) berries (204g/10MJ)		
				20		25%	25	M1 low PUFAs (3,1%) low in vegetables 167g/10MJ)		
				19		26%	23	M2 low PUFAs (3,2%) high in vegetables (440g/10MJ) berries (204g/10MJ)		
			CD	19		21%	32	Control Group		
Chung et al., 2022 (18)	Parallel	24	TD	16	4	37%	70	Anti-inflammatory Diet Group	CRP TNF-a MetS Component	↔ ns/Δ-60% vs -30% ↔ ns/Δ -5.47 vs -31% ↓ (p < 0.05)
			CD	15	4	60%	72	usual diet		
Schioldan et al., 2017 (19)	Cross over	28	WSD	19	4	68%	58	18 g/day of DF,4g from arabinoxylan, 3g from RS	IL-6 hsCRP IL-1RA Adiponectin LDL	↔ (p=0.640)/Δ0% vs -3% ↔ (p=0.16)/Δ-3% vs -15% ↔ (p=0.13)/Δ-11% vs 11% ↔ (p=0.87) ↓ (p<0.05)
			HCD		4			61g of DF, arabinoxylan (16 g/day) RS (21 g/day)		
Jenkins et al., 2005 (20)	Parallel	27	CD	18	4	55%	46	CD	LDL CRP	↓ (p<0.05) (PF) ↓ (p<0.05)/Δ-21% vs -53% (PF)
			PF	16	4			plant sterols (1.0 g/1000 kcal), sn plant sterols (1.0 g/1000 kcal), soy protein (21.4 g/1000 kcal), viscous fibers (9.8 g/1000 kcal), and almonds (14 g/1000 kcal)		
			Statin	14	4			CD with 20 mg lovastatin (statin)		
Roager et al., 2019 (21)	Cross over single blinded	28	WG	50	8	36%	49	whole grain diet 179 ± 50 g/day of WG 33g of DF/day	Body weight CRP IL-6 IL1-b TNF-a Gut microbiome	↓ (p<0.05) ↓ (p =0.003)/Δ61% vs -31% ↓ (p =0.009)/Δ66% vs -12% ↓ (p = 0.008)/Δ100% vs -42% ↔ ns/Δ0% ↔ ns
			CD	50	8			refined grain diet 13 ± 10 g/day of WG 21 g of DF/day		
Boers et al., 2014 (22)	Parallel, stratified single blinded	31	CD	16	2	26%	53	14,6g of PUFAs 28g of DF	hsCRP TNF-a Triglycerides MetS caracteristics HDL	↔ (p=0.96)/Δ30% vs 5% ↔ (p=0.32)/Δ0% vs 2% ↓ (p=0.001) ↓ (p=0.01) ↑ (p<0.05)
			Paleo	18	2			19g of PUFAs 34g of DF		
Juraschek et al., 2021 (23)	Parallel	29	CD	204	4	44%	48	11g of DF 300mg of cholesterol 8% energy of PUFAs	hs-CRP cardiac parameters	↓ (p=0.03)/Δ-1% vs -14% ↓ (p<0.001)
			Dash Diet	208				DD 3 levels of sodium intake 32g of DF 150mg Cholesterol 8% energy of PUFAs		
Zade et al., 2016 (24)	Parallel	28	CD	30	8	50%	43	Control Diet	hs-CRP MDA GSH HOMA-IR TG GSH	↓ (p<0.05)/ Δ-6.4% vs -25% ↓ (p<0.05) ↓ (p<0.05) ↓ (p<0.05) ↓ (p<0.05) ↑ (p<0.05)
			Dash Diet	30			40	Dash Diet		
Meslier et al., 2020 (25)	Parallel	31	MD	43	8	48%	43	MD	Total cholesterol Faecalibacterium prausnitzii hs-CRP	↓ (p<0.05) ↑ (p<0.05) ↔ ns: Δ23% vs 3%
			CD	39				CD		

**Table 2:** Bioactive mix intervention studies investigated the impact on systemic inflammation, oxidative stress and cardiometabolic risk markers.

References	Design	BMI	Treatment	n	Length (weeks)	Proportion of male subjects	Age	Components of the dietary intervention	Response variables	Positive (↑). inverse (↓). and no (↔) statistically significant associations (pvalue)/Rate of change Δ placebo vs enriched
Tovar et al., 2012 (9, 26)	Cross over	28	MFD	44	4	18%	63	CD	hs-CRP LDL/HDL HbA1c apoB/apoA1 systolic blood pressure triglycerides total serum cholesterol Gut microbiota composition	↓ (p<0.05) Δ12% vs -29% ↓ p < 0.0001 ↓ p = 0.0013 ↓ p < 0.0001 ↓ p = 0.0123 ↓ p=0.0056 ↓ p<0.0001 NS
			CD					Dietary Fiber, PUFA, soybean and whole barley kernel products, almonds, stanols and a probiotic strain (Lactobacillus plantarum Heal19/DSM15313)		
Scotto di Palumbo et al., 2022 (27)	Parallel double blinded	25	placebo	16	24	94%	75	fruit juice placebo (PLA)	hsCRP	↔ (p=0.855) Δ-6% vs 10%
			SUPP	21				supplement (SUPP) LC n-3 PUFA (3000 mg as 1500 mg DHA and 1500 mg EPA). whey protein isolate (8 g). vitamin D3 (400 IU). and resveratrol (150 mg)		
Bakker et al., 2010 (28)	Cross over double blinded	29	AID capsules	36	5	100%	46	6.3 mg resveratrol, 94.5g green tea extract, 90.7 mg a-tocopherol 125 mg vitamin C, 380 mg EPA, 260 mg DHA 60 mg other PUFAs, 3.75 mg lycopene	CRP Endothelial function Adipose tissue inflammation Adiponectin	↔ (NS) ↓ (p<0.05) ↓ (p<0.05) ↓ (p<0.05)
			Placebo capsules					microcrystalline cellulose (Microz Food Supplements) and 1360 mg soy lecithin		
Peluso et al., 2012 (29)	Cross over double blinded	27	Juice	14	<1	86%	45	32 mg/L Anthocyanins 0.5 mg/L Hydroxycinnamates 2.5 mg/L Flavan-3-ols 20mg/L Flavonols	Cholesterol TG IL-6 excursions TNF-a excursions	↓ (p<0.001) ↓ (p < 0.05) ↓ (p<0.05)
			placebo	14				placebo		
Vitaglione et al., 2015 (30)	Parallel	29	WG	36	8	31%	40	polyphenols + cereal dietary fiber	IL-10 TNF-a Clostridium reduction	↑ (p<0.05) Δ-4.51% vs 51% ↓ (p<0.05) Δ2.74% vs -28.9% ↓ (p<0.05)
			CD	32		37%	37	refined wheat (RW)		
Arcusa et al., 2021 (31)	Cross over double blinded	24	high-polyphenol	92	16	49%	34	encapsulated nutraceutical	TNF-a sTNFR1 OxLDL CRP	↓ (p=0.609) ↓ (p<0.05) ↓ (p<0.001) ↓ (p<0.001)
			Placebo					Placebo		
Solà et al., 2012 (32)	Parallel double blinded	28	CD	28	4	40,70%	54	CD	Ox-LDL Apo B/Apo A hsCRP	↓ (p=0.01) C/LMN vs A ↓ (p=0.01) C/LMN vs A ↓ (p<0.05) LMN vs A Δ34%
			TI	28				cocoa (1g/day) +hazelnut cream (30 g/day)		
			TI	30				cocoa (1g/day) +hazelnut cream (30 g/day) +phytosterols (2 g/day)		
			TI	27				cocoa (1g/day) +hazelnut cream (30 g/day) +phytosterols (2 g/day) +soluble fiber (20g/day)		
Yu Jin et al., 2010 (33)	Parallel. double blinded	26	placebo	41	8	27%	22-55	Placebo	MCP-1 MIP-1b RANTES Superoxide dismutase	↓ (p<0.05) Δ4% vs -38% (FVB) ↓ (p<0.05) Δ3% vs -17% (FVB) ↓ (p<0.05) Δ4% vs -21% (FVB) ↓ (p=0.01)
			juice + berry powders	38		24%		juice powderconcentrate with added berry powder 7.5 mg b-carotene, 276 mg vitamin C, 72 mg vitamin E in theform, 780mg folate, 80 mg calcium		
			juice powder concentrate	38		24%		juice powder concentrate 7.5 mg b-carotene,234 mg vitamin C, 30 mg vitamin E 420mg folate, 60 mg calcium		
Ranaivo et al., 2022 (34)	Cross over double blinded	28	MultiFiber	39	16	43%	43	enriched bread 16.05 g of fiber mix	hs-CRP LBP/CD14 LDL cholesterol HOMA insulin Bacteroides vulgatus	↔ (NS)/Δ-26% vs 1.49% ↔ (NS) ↓ q<0.01 ↓ q<0.05 ↓ q<0.05 ↓ q<0.01
			CD				41	standard bread 5.55 g fiber mix		
Rabbani et al., 2021 (35)	Cross over double blinded	30	trans-resveratrol hesperetin combination	38	12	38%	45	trans-resveratrol hesperetin combination	Gene expression: MCP-1 COX-2 IL-8 CCL2 RAGE	↓ (p<0.05) ↓ (p<0.05) ↓ (p<0.05) ↓ (p<0.05) ↓ (p<0.05)
			placebo					Placebo		
Cheung et al., 2022 (36)	Parallel double blinded	30	Magnesium + vitamine D	21	12	50%	45	MagD; 360 mg magnesium glycinate + 1000 IU vitamin D3 daily	IL-6 MCP-1 CRP Adiponectine PTH Cardiac Markers	↔ (NS)/Δ3% vs -21% (MagD) ↔ (NS)/Δ-1% vs -6% (MagD) ↔ (NS)/Δ3% vs -7% (MagD) ↔ (NS) ↔ (NS) ↔ (NS)
			VitD	34		50%	43	(VitD; 1000 IU vitamin D 3 daily)		
			placebo	23		64%	41	placebo		

TI, Test Intervention; AID, Anti-Inflammatory Diet; CD, Control Diet; RS, Resistant Starch; RD, Refined Grain Diet; PUFAs, Poly-Unsaturated Fatty Acids; MetS, metabolic syndrome; ns, non significant; DF, Dietary Fiber. For each inflammatory parameter, rates of change in % (Δ) have been added, except for articles where this information is not available.

### 2.2 Studies’ description

The duration of the studies varied from less than a week to up to 26 weeks. Most of the studies were mixed gender except for the two studies by Juscelino Tovar et al. and Bakker et al. (16, 28). 4 studies were conducted in healthy subjects, 11 in obese subjects (12, 24, 27–30, 32–36), 15 in overweight subjects, 3 in metabolic syndrome subject, 1 in hyperlipidemic subjects (37), 1 in hypertensive subjects’ stage 1 (32),1 study on subjects with hepatic steatosis (35).

Of all the inflammatory parameters evaluated in the studies, the most frequently found were high-sensitivity C-reactive protein (hs-CRP) or CRP in 21 studies, TNF-a for 8 studies, IL-6 for 6 studies. Out of the 27 studies examined, 16 investigated multiple inflammatory endpoints, and every study explored metabolic parameters alongside at least one inflammatory endpoint. The results are then presented by type of dietary approaches (ID or BM).

## 3 Results of integrated diet interventions

### 3.1 Descriptions of intervention

Concerning integrated diet studies, 1 was defined as Mediterranean diet (MD), 4 were “whole grain” diet (WG), 4 were “Nordic Diet” (ND), 2 were Dietary Approaches to Stop Hypertension (DASH) diets, 1 was a “portfolio” (PF), 1 was a “healthy carbohydrate” diet (HCD), 1 was a “paleo” diet (paleo), and 2 were “anti-inflammatory” diets (AID) or “antioxidant-rich” interventions (**Tables 1**, **2**).

#### 3.1.1 Mediterranean Diet (MD) interventions

The MD consists mainly of traditional foods from countries bordering the Mediterranean Sea such as berry fruits, vegetables, cereals, nut and seeds while limiting refined products and associated added sugar as found in the studies of this review. The MD is one of the most studied diets for preventing both CVD and inflammation (38). Its bioactive components have been found to reduce the risk of CDs by lowering cardiac and blood sugar parameters and by limitating oxidative stress.

In the case of the selected intervention study (25), each participant in the MD group adhered to an individually tailored diet that maintained the daily energy and macronutrient intake of their habitual diet, ensuring a dietary pattern typical of the Mediterranean diet. This nutritional intervention lasted for 8 weeks among overweight or obese individuals at cardiometabolic risk who were otherwise healthy.

#### 3.1.2 Portfolio diet (PF) interventions

The PF diet is based on the use of a range of foods that are known to reduce blood cholesterol (39). Viscous dietary fiber, soy protein, plant sterols and nuts together form the basis of the PF diet. Thanks to its low-fat content and quality, whose benefits have been validated by international groups such as the Canadian Cardiovascular Society for benefits on cardiovascular and metabolic risks (40). This diet has been also shown to improve LDL cholesterol for the prevention of cardio metabolic risk. Only one PF study investigating its impact on inflammatory profile was found (20).

#### 3.1.3 Whole grain (WG) Diet interventions

Whole grain consumption has been shown to reduce the risk of coronary heart disease, cardiovascular disease, cancer and the development of metabolic disorders such as diabetes (22). Whole grain are rich in bioactive compounds such as dietary fiber, antioxidants and phytochemicals, which have anti-inflammatory properties. Consuming whole grain has been shown to help reduce levels of inflammatory markers such as CRP and IL-6 (41).

For all 4 studies identified in the present review (14–16, 21), the level of WG that reached the interventions was higher than the USDA recommended level as described in Kirwan et al. study (14).

#### 3.1.4 Nordic Diet (ND) interventions

As for the PF diet, the ND presents similarities with the MD. Both diets are based on the daily use of fruits, vegetables, oil, fish, and restrict the use of saturated fats from milk or red meat (42). ND is based primarily on the use of berries, which contain a large amount of polyphenols. Both diets use seasonal products with a plant-based nutritional base (42). The main difference with MD is the oil origin; MD is focus on the use of olive oil whereas the ND will contain rapeseed oil. The Nordic food model has been shown to improve certain cardiac parameters such as blood pressure and certain blood lipid markers, making it a recognized model for improving cardiometabolic health (43). It has also been associated with a decrease in inflammatory markers such as C-reactive protein (CRP) and interleukin-6 (IL-6) (44). In our selection, four articles investigated the effects of the ND in healthy individuals or those at cardiometabolic risk over intervention periods ranging from 6 to 26 weeks (10–13).

#### 3.1.5 Paleolithic Diet (Paleo) interventions

The Paleo diet is a dietary approach based on the prehistoric food consumption pattern, emphasizing the consumption of unprocessed foods. This diet consists mainly of lean meats but also fish, and low-carbohydrate intake and does not allow the consumption of refined products such as oils or dairy products from industry (44). The Paleolithic diet, as reported by Ehsan Ghaedi et al., demonstrated short-term improvements in metabolic syndrome components (44).

Moreover, Ehsan Ghaedi et al. showed the role of this diet in decreasing CRP in relation to other metabolic risk factors in a meta-analysis (44). Among the articles selected, only one study was conducted with Paleo diet, on subjects with metabolic syndrome, with a duration of intervention lasting two weeks (22).

#### 3.1.6 Dietary approach to stop hypertension (DASH) Diet interventions

The DASH diet is rich in fruits, vegetables, poultry, fish, oilseeds, milk and low-fat dairy products (45). The DASH diet has historically demonstrated cardiac and metabolic benefits in normal and hypertensive subjects compared to a typical American diet, leading to a national recommendation of this diet (45). The DASH diet has exhibited efficacy in reducing levels of these primary indicators of inflammation, indicating its potential as an anti-inflammatory intervention (46). Furthermore, the DASH diet has been shown to effectively decrease inflammation markers linked to obesity, such as hs-CRP levels, when compared to unhealthy or customary diet (46). These findings support the notion that the DASH diet may serve as a valuable dietary intervention for mitigating low-grade inflammation, thereby contributing to improved overall health outcomes (46). Within our article selection, two DASH diet studies were conducted in subjects with overweight or moderate obesity for a duration of 4 to 8 weeks of intervention (23, 24).

### 3.2 Results of integrated diets interventions

#### 3.2.1 Inflammation parameters

In 4 out of 16 identified ID studies, significant improvements were observed in the subjects’ inflammatory profile as assessed by CRP levels. Studies that contributed to a decrease in circulating CRP concentrations from Roager et al. (21), Poulsen et al. (13) were based on intervention with a high dietary fiber intake (>33g). The studies that demonstrated improvements in hs-CRP levels specifically pertained to the DASH Diet studies conducted on individuals at cardiometabolic risk (23, 24). In the 4 identified studies examining IL-6, one demonstrated a significant improvement in this parameter (21).

In the Vanegas study, the WG intervention demonstrated a significant decrease in TNF-a levels (15). In contrast, the study conducted by Inge Boers, investigating the Paleo diet, showed no significant impact on hs-CRP and TNF-a levels in individuals with metabolic syndrome during a 2-week intervention period (22).

#### 3.2.2 Oxidative stress parameters:

Among all the investigated ID studies, 1 study have examined the effects of integrated nutritional interventions on oxidative stress parameters. Zade et al. (24) conducted a 60-day intervention with a MD diet and observed changes in inflammation and oxidative stress parameters (GSH). Serum levels of the pro-inflammatory marker, hs-CRP, were significantly reduced (p<0.05), while serum levels of the antioxidant enzyme were significantly increased in overweight subjects.

#### 3.2.3 Metabolic parameters

With the exception of one study, all ID interventions demonstrated benefits on metabolic or anthropometric parameters regardless of the intervention type, with only 31% of total ID studies showing a concurrent improvement in the inflammatory profile. Regarding metabolic parameters, from the 15 studies that reported both inflammation and metabolic parameters, only 5 showed significant parallel effect on these parameters (12, 13, 15, 23, 24). Regarding anthropometric parameters, the sole study demonstrating weight reduction was conducted by Poulsen et al. after 24-week of implementing a ND in obese subjects (13). In terms of insulin sensitivity and glycemic markers, Fritzen et al. and Zade et al. demonstrated an improvement in HOMA-IR following 26 and 8 weeks of the ND or DASH diet, respectively, in obese or overweight individuals, accompanied by a concomitant reduction in CRP in the latter study (11, 24). Lipid markers, such as triglycerides, were reduced in the Paleo and DASH diet studies respectively (22, 24).

#### 3.2.4 Inflammation and gut microbiota

Only 3 studies among our selected ID articles investigated the influence of WG diet and MD on the gut microbiota (15, 21, 25). Vanegas et al. revealed no significant alterations in Firmicutes, Bacteroidetes, and other microbial populations, nor in alpha diversity following the intervention (33) as did the study by Roager et al, which showed no change associated with the WG intervention (21). However, the study have demonstrated a significant change in the number of immune effectors (p<0.05) after a 6-week intervention with a WG diet in obese individuals. Meslier et al. have shown the impact of the MD intervention, notably an increase in the abundance of the species Faecalibacterium prausnitzii (25).

## 4 Bioactive mix interventions

### 4.1 Description of bioactive mix interventions

11 RCT BM studies focusing on the impact of a combination of two or more multifunctional bioactive compounds, included within a standard isocaloric diet, were integrated in this review.

Within BM studies, the most frequent combinations of bioactive compounds were vitamins with polyphenols as well as dietary fiber and polyphenols. Full details of each BM study are represented in **Table 2**, which summarizes the descriptions and types of interventions with the quality and quantity of the studied bioactive compounds.

8 studies have tested polyphenols of different classes in juice or concentrated powder form (27–33, 35) 3 BM studies have tested the impact of dietary fiber in soluble or integrated within a food (30, 32, 34). PUFAs are included in 2 BM studies in association with other bioactive compounds such as dietary fiber or polyphenols (27, 28). Of the two studies, lipids were mainly PUFAs mainly containing EPA and DHA always associated with polyphenols as well as a mix of vitamins for which the quantities are different between the two study types (**Tables 1**, **2**). The efficacy of various vitamins was assessed in three interventional studies, specifically investigating the effects of vitamins or combined with polyphenols or omega-3 fatty acids (27, 33, 36).

Among the studied inflammatory parameters, the distribution was very heterogeneous compared to the ID studies with CRP and IL-6 being the most represented markers.

### 4.2 Results of bioactive mix interventions

#### 4.2.1 Bioactive mix: inflammation parameters:

Among the seven selected BM studies that examined (hs-) CRP, three showed a significant decrease compared to the control diet in healthy or hypertensive subjects (31, 32). The first one have tested the effect of a mix of polyphenols in healthy subjects (31) and the second one have tested polyphenols, phytosterol and dietary fiber in hypertensive subjects (32) during 16 and 28 weeks of intervention respectively. Three studies showed a decrease in both circulating pro-inflammatory cytokines such as IL-6/17, TNF-a and CRP (29–31) with multifunctional BM interventions enriched in polyphenols, with or without dietary fiber all carried out in subjects at cardiometabolic risk, except for the study by Yu Jin et al. The only screened study that have tested two chemokines showed an improvement in RANTES after 8 weeks of polyphenol-rich fruit juice intervention and a significant decrease of MCP-1 in overweight subjects (33). The second study that investigated an effect on TNF-a, is the study by Vitaglione et al. that showed a benefit after 8 weeks of intervention with fiber-bound polyphenols (30). In the study of Tovar et al. (9), the BM intervention significantly reduced hs-CRP before and after adjustment for weight changes.

#### 4.2.2 Inflammation and oxidative stress:

Yu Jin et al. showed a positive impact of an antioxidant juice intervention on markers of oxidative stress with the bioactive mixture (antioxidant juice): a significant decrease in the enzymatic activity of superoxide dismutase, a marker in the production of free radicals on both healthy and at cardiometabolic risk subjects (33). This study, which investigated oxidative stress, also observed a joint improvement in RANTES and MCP-1 inflammatory parameters.

#### 4.2.3 Inflammation and metabolic parameters

Out of all these studies, eight studies have investigated metabolic parameters, including analyses of lipid metabolism, carbohydrate metabolism, as well as anthropometric parameters, alongside inflammatory parameters. The studies conducted by Arcusa et al. and Solà et al. (31, 32) demonstrated effects on both metabolic markers and inflammation. Arcusa et al.’s study notably revealed an impact on C-reactive protein in relation to oxidized low-density lipoprotein (ox-LDL) after a 16-week intervention with a high-polyphenolic nutraceutical in healthy subjects. Solà et al.’s study showed a benefit on high-sensitivity CRP after a 4-week intervention with polyphenol phytosterols and dietary fiber in stage 1-hypertensive subjects. The study by Baker et al. showed a beneficial impact of a mixture of polyphenols and vitamins on adiponectin in overweight and obese subjects after 5 weeks of intervention with no effect on inflammatory marker CRP (28). In addition, the study by Ranaivo et al. showed an improvement in LDL, HOMA and insulin parameters after 8 weeks of intervention with a multifiber bread with no effect on inflammatory marker hs-CRP (34). Tovar et al. study (9) showed multiple improvement in metabolic parameters notably in serum cholesterol, LDL/HDL, apoB/apoA1, HbA1c, and systolic blood pressure after 4 weeks of low glycemic impact meals, antioxidant-rich foods, oily fish as source of long-chain omega-3 fatty acids, dietary fibers and a probiotic strain. Finally, the only study that did not show significant effects on inflammatory or metabolic parameters was the study by Scotto di Palumbo et al. that tested an intervention with omega 3, polyphenols and vitamins in overweight subjects after 24 weeks of intervention.

#### 4.2.4 Inflammation and gut microbiota

Three studies have investigated the impact of BM interventions on microbiota abundance, with only one also assessing microbiota function simultaneously. Ranaivo et al. (34) examined the effect of a multifiber bread intervention on overweight or obese individuals. The multifiber bread intervention resulted in a significant alteration in the diversity of bacteria, specifically Bacteroides vulgatus (q < 0.1), when compared to the lower dietary fiber control bread after 16-week intervention. The second study demonstrating changes in the microbiota composition was conducted by Vitaglione et al. (30), revealing a reduction in TNF-a levels correlated with an increase in Bacteroides and Lactobacillus.

The study of Tovar et al. (26) did not significantly modify the gut microbiota composition at phylum or genus taxonomic levels.

## 5 Discussion

In this review, we examined the influence of combining multiple dietary compounds on low-grade inflammation, which play a key role in the development of metabolic alterations, particularly in at-risk individuals. Our findings reveal that RCT-based multifunctional dietary interventions have differential effects on managing low-grade inflammation depending not solely on the bioactive compounds content but also on the explored inflammatory markers, which demonstrated a large heterogeneity among studies. Diet-induced improvements in inflammatory profile were not specifically associated with alterations of cardiometabolic parameters or gut health.

First, we focused on BM studies, combining 2 or more multifunctional bioactive compounds, among which 72% demonstrated a beneficial effect on at least one inflammatory marker. The majority of studies showing benefits on the inflammatory axis involved mix of polyphenols or in combination with PUFAs or dietary fiber. Polyphenols, specifically flavonoids, are known to regulate the expression and production of cytokines such as IL-1b, TNF-a, IL-6, IL-8, and to prevent and treat intestinal inflammation through the modulation of Treg cell activity and promotion of beneficial microbiota proliferation within the intestinal environment (47, 48). Moreover, procyanidins have been acknowledged for their capacity to regulate the immune system, particularly through the inhibition of pro-inflammatory cascades such as NF-κB (nuclear factor-kappa B) and MAPK (Mitogen-activated protein kinases) (48), thereby mitigating the generation of pro-inflammatory mediators which aligns with the observed effects in the reviewed studies. That suggest that the potential multi-level impact of polyphenols on inflammation and cardiometabolic health. Presently, the main classes of polyphenols showing an impact on inflammatory markers are flavonoids and resveratrol (29, 32, 33). Concomitantly, we also observed in the studies by Peluso et al., Solà et al. and Jin et al. a beneficial role on the production of pro-inflammatory markers. The supplementation with polyphenols also showed beneficial effects when tested with dietary fiber, as in the study by Rosa Solà et al. (32). Dietary fiber have been shown to reduce the production of pro-inflammatory cytokines, more particularly resistant starch (49). Dietary fiber interact with gut microbiota and the resulting production of short chain fatty acids (SCFA) may improve gut permeability and alter the immune system through the activation of G protein-coupled receptors-related signalling pathways, which modulate the inhibition of the production of inflammatory cytokines (50). However, in the study by Ranaivo et al, a mix of dietary fibers did not significantly alter CRP or endotoxemia in at-risk individuals despite improvements in cardiometabolic parameters (34).

As for studies testing PUFAs, both were associated with other components such as polyphenols or dietary fiber (22, 28) and none of the combination showed an impact of inflammation as assessed solely by CRP, despite beneficial effects on insulin sensitivity and lipid metabolism. Dietary fats can impact inflammatory profile by modulating both pro-inflammatory and anti-inflammatory processes (51). Presently both Scotto et al. and Bakker et al. studies used supplementation with DHA (docosahexaenoic acid) and EPA (eicosapentaenoic acid), precursors of anti-inflammatory eicosanoids previously shown to reduce CRP in subjects presenting with dyslipidaemia or higher baseline inflammatory status, as synthetized in Guo’s meta-analysis (52).

As for ID studies, 31,25% have demonstrated an improvement in inflammatory parameters, with a parallel metabolic effect in more than half of them. The common denominator among the ID diets, Mediterranean, Nordic, DASH and Paleo diets, which have shown a beneficial effect on low-grade inflammation markers is the combination of polyphenols, dietary fiber, vitamins and omega-3 fatty acids. The potential impact of Mediterranean diet components on cardiometabolic and inflammation markers has been extensively studied and reviewed through their actions on adipocytes and on the innate immune system (53). Published works have demonstrated the efficacy of the Nordic food model in enhancing specific cardiovascular indicators but no impact on inflammatory markers such as CRP, TNF-a, and IL-6 as previously reviewed (43). Consistently in our review, the ND studies showed a beneficial impact on the cardiometabolic profile and only half of them an improvement in CRP, IL-6 but no other interleukins. The DASH diet has been shown to effectively reduce obesity-related markers of inflammation, such as hs-CRP levels, compared to usual diets (24). Of the two studies that tested this DASH diet, both showed a significant improvement in the inflammatory profile of hs-CRP, with the study by Zade et al. showing a joint improvement in oxidative stress and metabolic parameters (23, 24). Concerning the Paleo diet, the results were more mixed, with an improvement in markers of metabolic syndrome but no improvement in the inflammatory profile (22), contrary to what was shown in the meta-analysis by Ehsan Ghaedi et al. (44). Notably, the research discussed in this review primarily emphasized well-established inflammation markers such as CRP, IL-6, and TNF-a. Moreover, there has been limited exploration of a broader spectrum of markers associated with low-grade inflammation or oxidative stress, as only half of the RCTs reported a single inflammatory or oxidative stress marker.

Considering the highly intricate nature of inflammation processes mediated by diverse cellular actors, a comprehensive examination of pro- and anti-inflammatory cytokines becomes imperative for comprehending the specific impact of nutritional interventions on distinct cellular constituents. Interestingly, when several markers were analyzed, the impact of dietary intervention was similar. Furthermore, as observed in this review, only a few anti-inflammatory markers have been investigated within the studies, compared to pro-inflammatory markers. The balance between pro- and anti-inflammatory markers represents the global inflammatory state and needs to be further investigated. For example, lipid mediators derived from omega-3 polyunsaturated fatty acids, such as resolvins, play a crucial role in resolving inflammation. Their ability to regulate immune and inflammatory responses makes them potentially beneficial in the treatment of chronic inflammatory diseases, but they remain largely unexplored in nutritional interventions (51).

To address the global inflammatory status, an alternative approach is to use composite inflammatory scores derived from multiple inflammatory markers to evaluate the effects of interventional studies by estimating overall inflammatory status. Such strategies could prove useful for improving sensitivity to detect changes following nutritional intervention, particularly in healthy subjects (54).

Moreover, although the relationship between diet, gut microbiota and inflammation is a crucial, few studies explored specific microbial markers, originating from the gut gram negative bacteria, identified as being associated with inflammation and metabolism, such as flagellin or lipopolysaccharides (LPS), also known as endotoxins (55). These endotoxins can trigger inflammation by binding to immune receptors, such as Toll-like receptor 4 (TLR-4). Only one study on the impact of a mix of dietary fibers on gut microbiota composition, gut health and metabolic profile investigated markers of metabolic endotoxemia but no effect was detected (34). Interestingly, alterations in the composition of the microbiota may also impact inflammatory status, potentially through the presence of certain beneficial bacteria, such as Faecalibacterium prausnitzii (25).

It should be kept in mind that these cytokines are present at the systemic level when homeostasis is strongly altered and it has been proposed that dietary challenges stressing homeostasis could be a more relevant condition to address the dynamic impact of food items on inflammatory status and may increase the robustness of the studies carried out in particular in healthy or at-risk subjects (1, 6, 55). Van den Brink et al. argue for the use of dynamic challenges to complement fasting measurements information (7). Indeed, the postprandial phase appears to be a complementary period providing additional information for detecting early alterations in metabolism and inflammatory status, particularly during nutritional interventions with diverse bioactive compounds targeting a significant number of pathways. In this sense, Emerson et al. reviewed the magnitude and interest of several inflammatory markers assessment after a high-fat meal challenge and concluded that beyond CRP and TNF-a, not responsive in the postprandial phase, other inflammatory markers, such as leukocyte-bound markers should be further investigated (56).

Comprehensive fasting and postprandial evaluation could facilitate a nuanced understanding of the complex interaction between bioactive compounds, the food matrix and their cumulative impact on inflammatory responses (57, 58). Finally dietary scores have been developed such as the Dietary Inflammatory Index that report the potential inflammatory effect of foods could play a significant role in improving our understanding of the mechanistic effects of bioactive compounds, both when administered individually and within a dietary matrix, in modulating specific inflammatory markers (59). Such information may improve researchers’ ability to design precise and effective nutritional interventions, designed to target specific inflammatory pathways (60). Using indexes such as the DII could allow to manage diet by potentiating the synergistic interactions contained in all the available foods in order to effectively prevent the onset of the disease.

### 5.1 Strengths and limitations

We conducted a comprehensive analysis based on strict selection criteria of targeted randomized controlled trials, primarily focused on individuals with cardiometabolic risk and combination of multifunctional compounds within usual diet. It is important to acknowledge that variations in study outcomes may arise from divergent experimental designs, particularly in terms of intervention duration, participants, types of bioactive compounds used, and parameters studied. Moreover, since these studies did not extensively explore the influence of the microbiota and primarily focused on examining fasting markers, it is plausible that broader effects on cardiometabolic health may have been overlooked. Therefore, a more comprehensive mechanistic understanding of low-grade inflammation would strongly support the adoption of standardized markers to effectively isolate the individual effects of each intervention. We need to acknowledge that for many studies inflammatory markers were not the primary outcome thus some impact could have been underpowered-and under-estimated. Moreover, the external validity of present findings is limited to healthy and at-risk individuals.

## 6 Conclusion

Our review demonstrates that multifunctional interventions, whether integrated into a diet or as bioactive mix supplements, exhibit diverse impacts on low-grade inflammation markers, contingent on specific ingredient combinations. Although TNF-alpha and CRP are the most commonly reported, notable finding is the considerable heterogeneity in the inflammatory markers studied across various trials that limits rigourous comparisons between combinations. Significant improvements in inflammatory profiles from multifunctional interventions do not consistently correlate with enhancements in cardiometabolic profiles. The balance between pro- and anti-inflammatory markers emerges as crucial, emphasizing the need for multiple markers analysis or composite inflammatory scores to comprehensively evaluate the overall impact of nutritional interventions.

Further research is warranted to assess the effectiveness of multifunctional dietary interventions on specific inflammatory markers, providing deeper insights into the links between low-grade inflammation and other cardiometabolic risk factors, such as intestinal inflammation or postprandial inflammatory dynamics.

## Author contributions

HH: Writing – original draft. AA: Writing – review & editing. MM: Conceptualization, Writing – review & editing. SV: Validation, Writing – review & editing. CC: Writing – review & editing. JN: Conceptualization, Investigation, Validation, Writing – original draft, Writing – review & editing.

## References

[1] Minihane AM Vinoy S Russell WR Baka A Roche HM Tuohy KM. Low-grade inflammation, diet composition and health: current research evidence and its translation. Br J Nutr (2015) 114(7):999–1012. doi: 10.1017/S0007114515002093 26228057 PMC4579563

[2] Hotamisligil GS. Inflammation, metaflammation and immunometabolic disorders. Nature (2017) 542(7640):177–85. doi: 10.1038/nature21363 28179656

[3] Bulló M Casas-Agustench P Amigó-Correig P Aranceta J Salas-Salvadó J. Inflammation, obesity and comorbidities: the role of diet. Public Health Nutr (2007) 10:1164–72. doi: 10.1017/S1368980007000663 17903326

[4] Koelman L Egea Rodrigues C Aleksandrova K. Effects of dietary patterns on biomarkers of inflammation and immune responses: A systematic review and meta-analysis of randomized controlled trials. Advances in nutrition (Bethesda, Md.) (2023) 13(1):.101–15. doi: 10.1093/advances/nmab086 PMC8803482 34607347

[5] Garcia-Arellano A Ramallal R Ruiz-Canela M Salas-Salvadó J Corella D Shivappa N. Dietary inflammatory index and incidence of cardiovascular disease in the PREDIMED study. Nutrients (2015) 7:4124–38. doi: 10.3390/nu7064124 PMC4488776 26035241

[6] Rochlani Y Pothineni NV Kovelamudi S Mehta JL. Metabolic syndrome: pathophysiology, management, and modulation by natural compounds. Ther Adv Cardiovasc Dis (2017) 11:215–25. doi: 10.1177/1753944717711379 PMC5933580 28639538

[7] van den Brink W van Bilsen J Salic K Hoevenaars FPM Verschuren L Kleemann R. Current and future nutritional strategies to modulate inflammatory dynamics in metabolic disorders. Front Nutr (2019) 6. doi: 10.3389/fnut.2019.00129 PMC6718105 31508422

[8] Calder PC Ahluwalia N Brouns F Buetler T Clement K Cunningham K. Dietary factors and low-grade inflammation in relation to overweight and obesity. Br J Nutr (2011) 106:S5–78. doi: 10.1017/S0007114511005460 22133051

[9] Tovar J Nilsson A Johansson M Ekesbo R Aberg A-M Johansson U. A diet based on multiple functional concepts improves cardiometabolic risk parameters in healthy subjects. Nutr Metab (2012) 9:29. doi: 10.1186/1743-7075-9-29 PMC3361470 22472183

[10] Adamsson V Reumark A Fredriksson I-B Hammarström E Vessby B Johansson G. Effects of a healthy Nordic diet on cardiovascular risk factors in hypercholesterolaemic subjects: a randomized controlled trial (NORDIET). J Intern Med (2011) 269:150–9. doi: 10.1111/j.1365-2796.2010.02290.x 20964740

[11] Fritzen AM Lundsgaard A-M Jordy AB Poulsen SK Stender S Pilegaard H. New nordic diet-induced weight loss is accompanied by changes in metabolism and AMPK signaling in adipose tissue. J Clin Endocrinol Metab (2015) 100:3509–19. doi: 10.1210/jc.2015-2079 26126206

[12] Uusitupa M Hermansen K Savolainen MJ Schwab U Kolehmainen M Brader L. Effects of an isocaloric healthy Nordic diet on insulin sensitivity, lipid profile and inflammation markers in metabolic syndrome – a randomized study (SYSDIET). J Intern Med (2013) 274:52–66. doi: 10.1111/joim.12044 23398528 PMC3749468

[13] Poulsen SK Due A Jordy AB Kiens B Stark KD Stender S. Health effect of the New Nordic Diet in adults with increased waist circumference: a 6-mo randomized controlled trial. Am J Clin Nutr (2014) 99:35–45. doi: 10.3945/ajcn.113.069393 24257725

[14] Kirwan JP Malin SK Scelsi AR Kullman EL Navaneethan SD Pagadala MR. A whole-grain diet reduces cardiovascular risk factors in overweight and obese adults: A randomized controlled trial. J Nutr (2016) 146:2244–51. doi: 10.3945/jn.116.230508 PMC5086786 27798329

[15] Vanegas SM Meydani M Barnett JB Goldin B Kane A Rasmussen H. Substituting whole grains for refined grains in a 6-wk randomized trial has a modest effect on gut microbiota and immune and inflammatory markers of healthy adults. Am J Clin Nutr (2017) 105:635–50. doi: 10.3945/ajcn.116.146928 PMC5320415 28179226

[16] Tovar J Nilsson A Johansson M Björck I. Combining functional features of whole-grain barley and legumes for dietary reduction of cardiometabolic risk: a randomised cross-over intervention in mature women. Br J Nutr (2014) 111:706–14. doi: 10.1017/S000711451300305X 24063257

[17] Freese R Vaarala O Turpeinen AM Mutanen M. No difference in platelet activation or inflammation markers after diets rich or poor in vegetables, berries and apple in healthy subjects. Eur J Nutr (2004) 43:175–82. doi: 10.1007/s00394-004-0456-4 15168040

[18] Chung H-K Kim JM Choi A Ahn CW Kim YS Nam JS. Antioxidant-rich dietary intervention improves cardiometabolic profiles and arterial stiffness in elderly Koreans with metabolic syndrome. Yonsei Med J (2022) 63:26–33. doi: 10.3349/ymj.2022.63.1.26 34913281 PMC8688374

[19] Schioldan AG Gregersen S Hald S Bjørnshave A Bohl M Hartmann B. Effects of a diet rich in arabinoxylan and resistant starch compared with a diet rich in refined carbohydrates on postprandial metabolism and features of the metabolic syndrome. Eur J Nutr (2018) 57:795–807. doi: 10.1007/s00394-016-1369-8 28070639

[20] Jenkins DJA Kendall CWC Marchie A Faulkner DA Wong JMW de Souza R. Effects of a dietary portfolio of cholesterol-lowering foods vs lovastatin on serum lipids and C-reactive protein. JAMA (2003) 290:502–10. doi: 10.1001/jama.290.4.502 12876093

[21] Roager HM Vogt JK Kristensen M Hansen LBS Ibrügger S Mærkedahl RB. Whole grain-rich diet reduces body weight and systemic low-grade inflammation without inducing major changes of the gut microbiome: a randomised cross-over trial. Gut (2019) 68:83–93. doi: 10.1136/gutjnl-2017-314786 29097438 PMC6839833

[22] Boers I Muskiet FAJ Berkelaar E Schut E Penders R Hoenderdos K. Favourable effects of consuming a Palaeolithic-type diet on characteristics of the metabolic syndrome: a randomized controlled pilot-study. Lipids Health Dis (2014) 13:160. doi: 10.1186/1476-511X-13-160 25304296 PMC4210559

[23] Juraschek SP Kovell LC Appel LJ Miller ER Sacks FM Chang AR. Effects of diet and sodium reduction on cardiac injury, strain, and inflammation: the DASH-sodium trial. J Am Coll Cardiol (2021) 77(21):2625–34. doi: 10.1016/j.jacc.2021.03.320 PMC8256779 34045018

[24] Razavi Zade M Telkabadi MH Bahmani F Salehi B Farshbaf S Asemi Z. The effects of DASH diet on weight loss and metabolic status in adults with non-alcoholic fatty liver disease: a randomized clinical trial. Liver international : official journal of the International Association for the Study of the Liver (2016) 36(4):563–71. doi: 10.1111/liv.12990 26503843

[25] Meslier V Laiola M Roager HM De Filippis F Roume H Quinquis B. Mediterranean diet intervention in overweight and obese subjects lowers plasma cholesterol and causes changes in the gut microbiome and metabolome independently of energy intake. Gut (2020) 69:1258–68. doi: 10.1136/gutjnl-2019-320438 PMC7306983 32075887

[26] Marungruang N Tovar J Björck I Hållenius FF. Improvement in cardiometabolic risk markers following a multifunctional diet is associated with gut microbial taxa in healthy overweight and obese subjects. Eur J Nutr (2018) 57:2927–36. doi: 10.1007/s00394-017-1563-3 PMC6267413 29098426

[27] Scotto di Palumbo A McSwiney FT Hone M McMorrow AM Lynch G De Vito G. Effects of a long chain n-3 polyunsaturated fatty acid-rich multi-ingredient nutrition supplement on body composition and physical function in older adults with low skeletal muscle mass. J Diet. Suppl (2021) 0:1–16. doi: 10.1080/19390211.2021.1897057 33759678

[28] Bakker GC van Erk MJ Pellis L Wopereis S Rubingh CM Cnubben NH. An antiinflammatory dietary mix modulates inflammation and oxidative and metabolic stress in overweight men: a nutrigenomics approach. Am J Clin Nutr (2010) 91:1044–59. doi: 10.3945/ajcn.2009.28822 20181810

[29] Peluso I Villano DV Roberts SA Cesqui E Raguzzini A Borges G. Consumption of mixed fruit-juice drink and vitamin C reduces postprandial stress induced by a high fat meal in healthy overweight subjects. Curr Pharm Des (2014) 20:1020–4. doi: 10.2174/138161282006140220144802 23701571

[30] Vitaglione P Mennella I Ferracane R Rivellese AA Giacco R Ercolini D. Whole-grain wheat consumption reduces inflammation in a randomized controlled trial on overweight and obese subjects with unhealthy dietary and lifestyle behaviors: role of polyphenols bound to cereal dietary fiber. Am J Clin Nutr (2015) 101:251–61. doi: 10.3945/ajcn.114.088120 25646321

[31] Arcusa R Carrillo JÁ Xandri-Martínez R Cerdá B Villaño D Marhuenda J. Effects of a fruit and vegetable-based nutraceutical on biomarkers of inflammation and oxidative status in the plasma of a healthy population: A placebo-controlled, double-blind, and randomized clinical trial. Molecules (2021) 26:3604. doi: 10.3390/molecules26123604 34204618 PMC8231220

[32] Solà R Valls RM Godàs G Perez-Busquets G Ribalta J Girona J. Cocoa, hazelnuts, sterols and soluble fiber cream reduces lipids and inflammation biomarkers in hypertensive patients: a randomized controlled trial. PloS One (2012) 7:e31103. doi: 10.1136/gutjnl-2017-314786 22383996 PMC3287993

[33] Jin Y Cui X Singh UP Chumanevich AA Harmon B Cavicchia P. Systemic inflammatory load in humans is suppressed by consumption of two formulations of dried, encapsulated juice concentrate. Mol Nutr Food Res (2010) 54:1506–14. doi: 10.1002/mnfr.200900579 20425759

[34] Ranaivo H Thirion F Béra-Maillet C Guilly S Simon C Sothier M. Increasing the diversity of dietary fibers in a daily-consumed bread modifies gut microbiota and metabolic profile in subjects at cardiometabolic risk. Gut Microbes (2022) 14:2044722. doi: 10.1080/19490976.2022.2044722 35311446 PMC8942430

[35] Rabbani N Xue M Weickert MO Thornalley PJ. Reversal of insulin resistance in overweight and obese subjects by trans-resveratrol and hesperetin combination-link to dysglycemia, blood pressure, dyslipidemia, and low-grade inflammation. Nutrients (2021) 13:2374. doi: 10.3390/nu13072374 34371884 PMC8308792

[36] Cheung MM Dall RD Shewokis PA Altasan A Volpe SL Amori R. The effect of combined magnesium and vitamin D supplementation on vitamin D status, systemic inflammation, and blood pressure: A randomized double-blinded controlled trial. Nutrition (Burbank, Los Angeles County, Calif.) (2022) 99-100L111674. doi: 10.1016/j.nut.2022.111674 35576873

[37] Jenkins DJ Kendall CW Marchie A Faulkner DA Wong JM de Souza R. Direct comparison of a dietary portfolio of cholesterol-lowering foods with a statin in hypercholesterolemic participants. Am J Clin Nutr (2005) 82(2):380–7. doi: 10.1093/ajcn.81.2.380 15699225

[38] Widmer RJ Flammer AJ Lerman LO Lerman A. The Mediterranean diet, its components, and cardiovascular disease. Am J Med (2015) 128:229–38. doi: 10.1016/j.amjmed.2014.10.014 PMC4339461 25447615

[39] Jenkins DJ Jones PJ Lamarche B Kendall CW Faulkner D Cermakova L. Effect of a dietary portfolio of cholesterol-lowering foods given at 2 levels of intensity of dietary advice on serum lipids in hyperlipidemia: a randomized controlled trial. JAMA (2011) 306(8):831–9. doi: 10.1001/jama.2011.1202 21862744

[40] Pearson GJ Thanassoulis G Anderson TJ Barry AR Couture P Dayan N. 2021 Canadian cardiovascular society guidelines for the management of dyslipidemia for the prevention of cardiovascular disease in adults. Can J Cardiol (2021) 37:1129–50. doi: 10.1016/j.cjca.2021.03.016 33781847

[41] Schwingshackl L Chaimani A Hoffmann G Schwedhelm C Boeing H. A network meta-analysis on the comparative efficacy of different dietary approaches on glycaemic control in patients with type 2 diabetes mellitus. European J Epidemiology (2018) 33(2):157–70. doi: 10.1007/s10654-017-0352-x PMC5871653 29302846

[42] Krznarić Ž Karas I Ljubas Kelečić D Vranešić Bender D. The mediterranean and nordic diet: A review of differences and similarities of two sustainable, health-promoting dietary patterns. Front Nutr (2021) 8:683678. doi: 10.3389/fnut.2021.683678 34249991 PMC8270004

[43] Ramezani-Jolfaie N Mohammadi M Salehi-Abargouei A. The effect of healthy Nordic diet on cardio-metabolic markers: a systematic review and meta-analysis of randomized controlled clinical trials. Eur J Nutr (2019) 58:2159–74. doi: 10.1007/s00394-018-1804-0 30128767

[44] Ghaedi E Mohammadi H Ramezani-Jolfaie N Malekzadeh J Hosseinzadeh M. Effects of a paleolithic diet on cardiovascular disease risk factors: A systematic review and meta-analysis of randomized controlled trials. Adv Nutr Bethesda Md (2019) 10:634–46. doi: 10.1093/advances/nmz007 PMC6628854 31041449

[45] Sacks FM Svetkey LP Vollmer WM Appel LJ Bray GA Harsha D. Effects on blood pressure of reduced dietary sodium and the Dietary Approaches to Stop Hypertension (DASH) diet. DASH-Sodium Collaborative Research Group. N Engl J Med (2001) 344:3–10. doi: 10.1056/NEJM200101043440101 11136953

[46] Soltani S Chitsazi MJ Salehi-Abargouei A. The effect of dietary approaches to stop hypertension (DASH) on serum inflammatory markers: A systematic review and meta-analysis of randomized trials. Clin Nutr Edinb. Scotl (2018) 37:542–50. doi: 10.1016/j.clnu.2017.02.018 28302405

[47] Grosso G Sahana GR Nagella P Joseph BV Alessa FM Al-Mssallem MQ. Flavonoids as potential anti-inflammatory molecules: A review. Molecules 27(9):2901. doi: 10.3390/molecules27092901 PMC9100260 35566252

[48] Shakoor H Feehan J Apostolopoulos V Platat C Al Dhaheri AS Ali HI. Immunomodulatory effects of dietary polyphenols. Nutrients (2021) 13(3):728. doi: 10.3390/nu13030728 33668814 PMC7996139

[49] Vahdat M Hosseini SA Khalatbari Mohseni G Heshmati J Rahimlou M. Effects of resistant starch interventions on circulating inflammatory biomarkers: a systematic review and meta-analysis of randomized controlled trials. Nutrition J (2020) 19(1):33. doi: 10.1186/s12937-020-00548-6 32293469 PMC7158011

[50] Grosso G Laudisio D Frias-Toral E Barrea L Muscogiuri G Savastano S. Anti-inflammatory nutrients and obesity-associated metabolic-inflammation: state of the art and future direction. Nutrients (2022) 14:1137. doi: 10.3390/nu14061137 35334794 PMC8954840

[51] Serhan CN Levy BD. Resolvins in inflammation: emergence of the pro-resolving superfamily of mediators. J Clin Invest (2018) 128(7):2657–69. doi: 10.1172/JCI97943 PMC6025982 29757195

[52] Guo X-F Li K-L Li J-M Li D. Effects of EPA and DHA on blood pressure and inflammatory factors: a meta-analysis of randomized controlled trials. Crit Rev Food Sci Nutr 59(20):3380–3393. doi: 10.1080/10408398.2018.1492901 29993265

[53] Tsigalou C Konstantinidis T Paraschaki A Stavropoulou E Voidarou C Bezirtzoglou E. Mediterranean diet as a tool to combat Inflammation and chronic diseases. An Overview. Biomedicines (2020) 8(7):201. doi: 10.3390/biomedicines8070201 32650619 PMC7400632

[54] van der Kolk BW Kalafati M Adriaens M van Greevenbroek MMJ Vogelzangs N Saris WHM. Subcutaneous adipose tissue and systemic inflammation are associated with peripheral but not hepatic insulin resistance in humans. Diabetes (2019) 68(12):2247–58. doi: 10.2337/db19-0560 31492661

[55] Jacobs DR Gross MD. Flagellin from gram-negative bacteria is a potent mediator of acute pulmonary inflammation in sepsis. Shock Augusta Ga (2003) 19:131–7. doi: 10.1097/00024382-200302000-00008 12578121

[56] Emerson SR Kurti SP Harms CA Haub MD Melgarejo T Logan C. Magnitude and timing of the postprandial inflammatory response to a high-fat meal in healthy adults: A systematic review. Advances in Nutrition (Bethesda, Md.) (2017) 8:213–25. doi: 10.3945/an.116.014431 PMC5347112 28298267

[57] Aguilera JM. The food matrix: implications in processing, nutrition and health. Crit Rev Food Sci Nutr (2019) 59:3612–29. doi: 10.1080/10408398.2018.1502743 30040431

[58] Jacobs DR Gross MD Tapsell LC. Food synergy: an operational concept for understanding nutrition. Am J Clin Nutr (2009) 89:1543S–8S. doi: 10.3945/ajcn.2009.26736B PMC2731586 19279083

[59] Shivappa N Steck SE Hurley TG Hussey JR Hébert JR. Designing and developing a literature-derived, population-based dietary inflammatory index. Public Health Nutr (2014) 17:1689–96. doi: 10.1017/S1368980013002115 PMC3925198 23941862

[60] Marx W Veronese N Kelly JT Smith L Hockey M Collins S. The dietary inflammatory index and human health: an umbrella review of meta-analyses of observational studies. Adv Nutr Bethesda Md (2021) 12:1681–90. doi: 10.1093/advances/nmab037 PMC8483957 33873204

